# Supplementary material for: Compounds from the Petroleum Ether Extract of Wedelia chinensis with Cytotoxic, Anticholinesterase, Antioxidant, and Antimicrobial Activities
Source: Molecules. 2023 Jan 13;28(2):793. doi: 10.3390/molecules28020793 (PMC9865212; doi:10.3390/molecules28020793)
Supplement: Supplementary file 1 [file molecules-28-00793-s001.zip › molecules-2059259-supplementary.pdf]

Supplementary Materials

Compounds from the Petroleum Ether Extract of *Wedelia chinensis* with Cytotoxic, Anticholinesterase, Antioxidant, and Antimicrobial Activities

Md. Abdur Rashid Khan [1], Md. Aminul Islam [2], Kushal Biswas [1,3], Md. Yusuf Al-Amin [1,4], Md. Salim Ahammed [1,5], Md. Imran Nur Manik [1,6], KM Monirul Islam [1], Md. Abdul Kader [1], A.H.M. Khurshid Alam [1], Shahed Zaman [2] and Golam Sadik [1,*]

[1] Department of Pharmacy, University of Rajshahi, Rajshahi 6205, Bangladesh
[2] Department of Chemistry, University of Rajshahi, Rajshahi 6205, Bangladesh
[3] Department of Biomedical and Nutritional Sciences, Zuckerberg College of Health Sciences, University of Massachusetts Lowell, Lowell, MA 01854, USA
[4] Purdue University Interdisciplinary Life Sciences Graduate Program, Purdue University, West Lafayette, IN 47907, USA
[5] Basic Biomedical Sciences, Sanford School of Medicine, University of South Dakota, Vermillion, SD 57069, USA
[6] Department of Bioscience, Graduate School of Science and Technology, National University Corporation Shizuoka University, 836 Ohyo, Suruga-ku, Shizuoka 422-8529, Japan
* Correspondence: gsadik2@ru.ac.bd

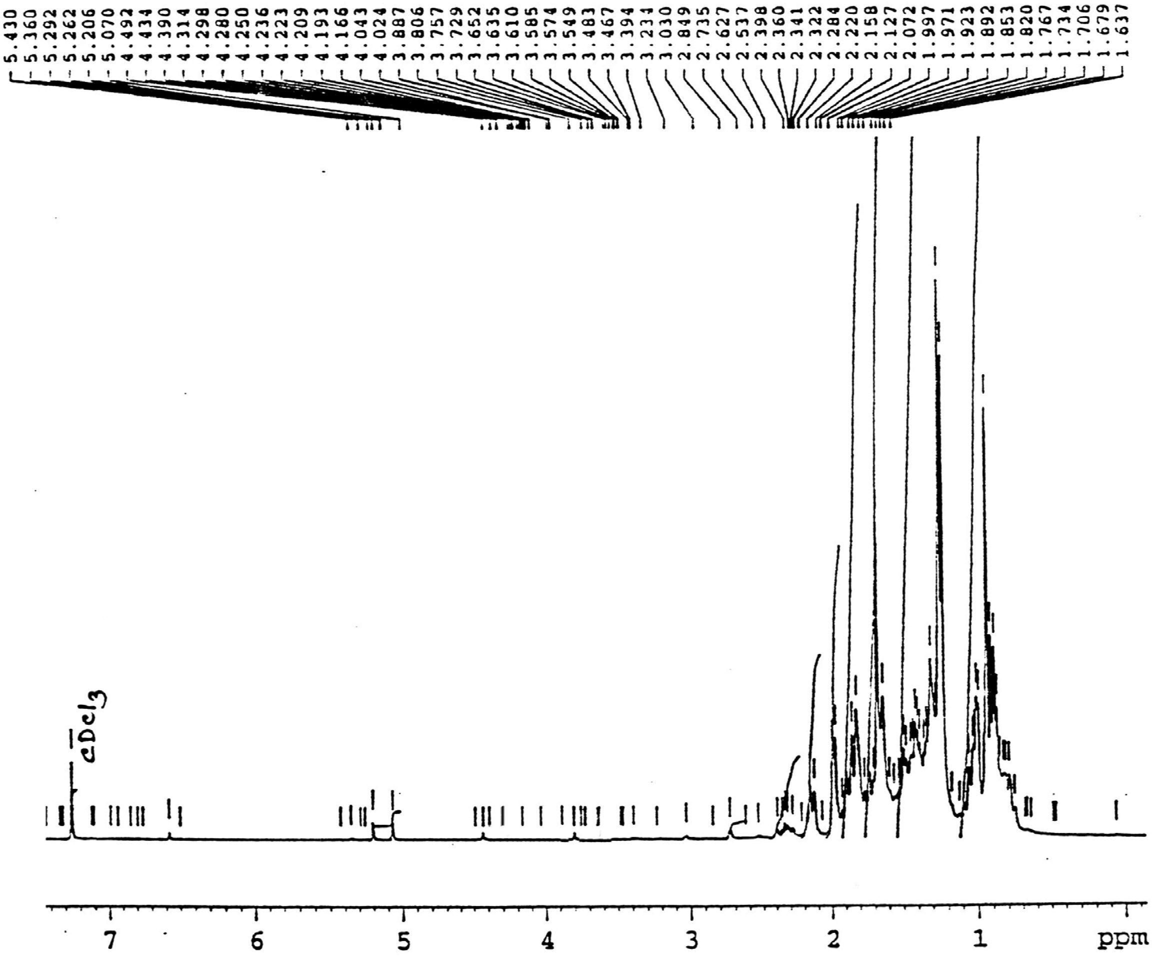

Figure S1. 1H NMR (400 MHz, CDCl3) spectrum of compound **1**.

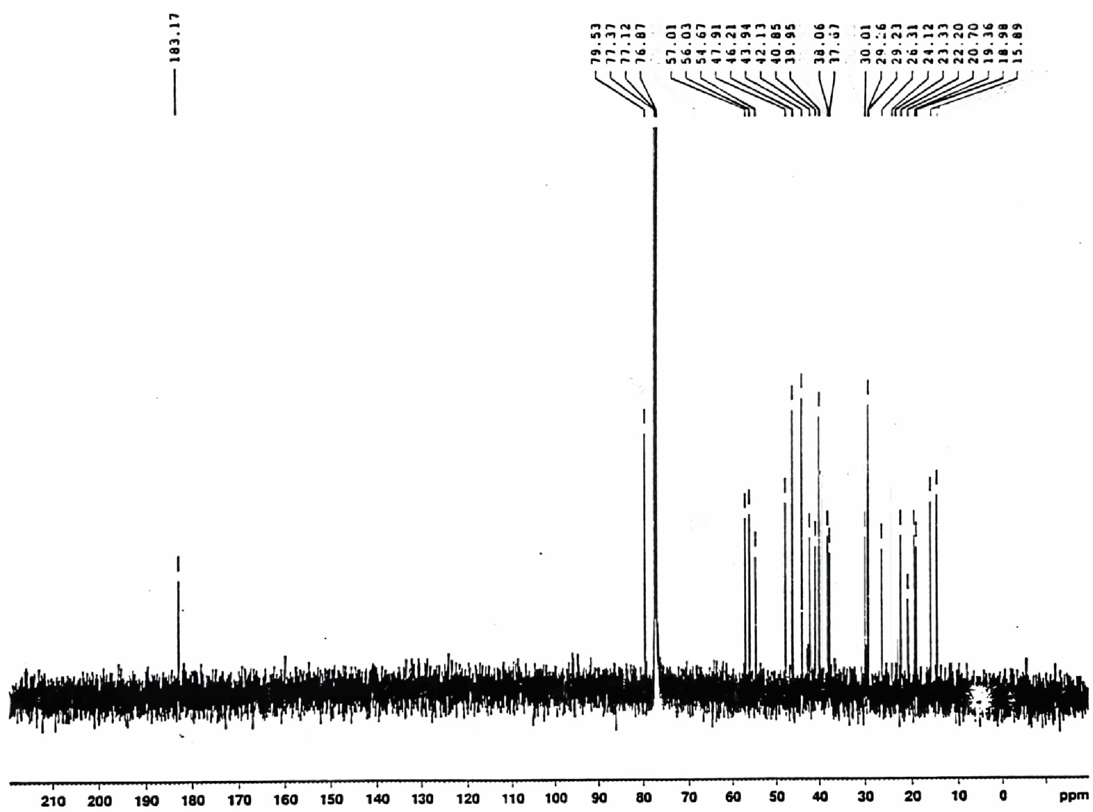

Figure S2.^{13}C NMR (100 MHz, CDCl3) spectrum of compound **1**.

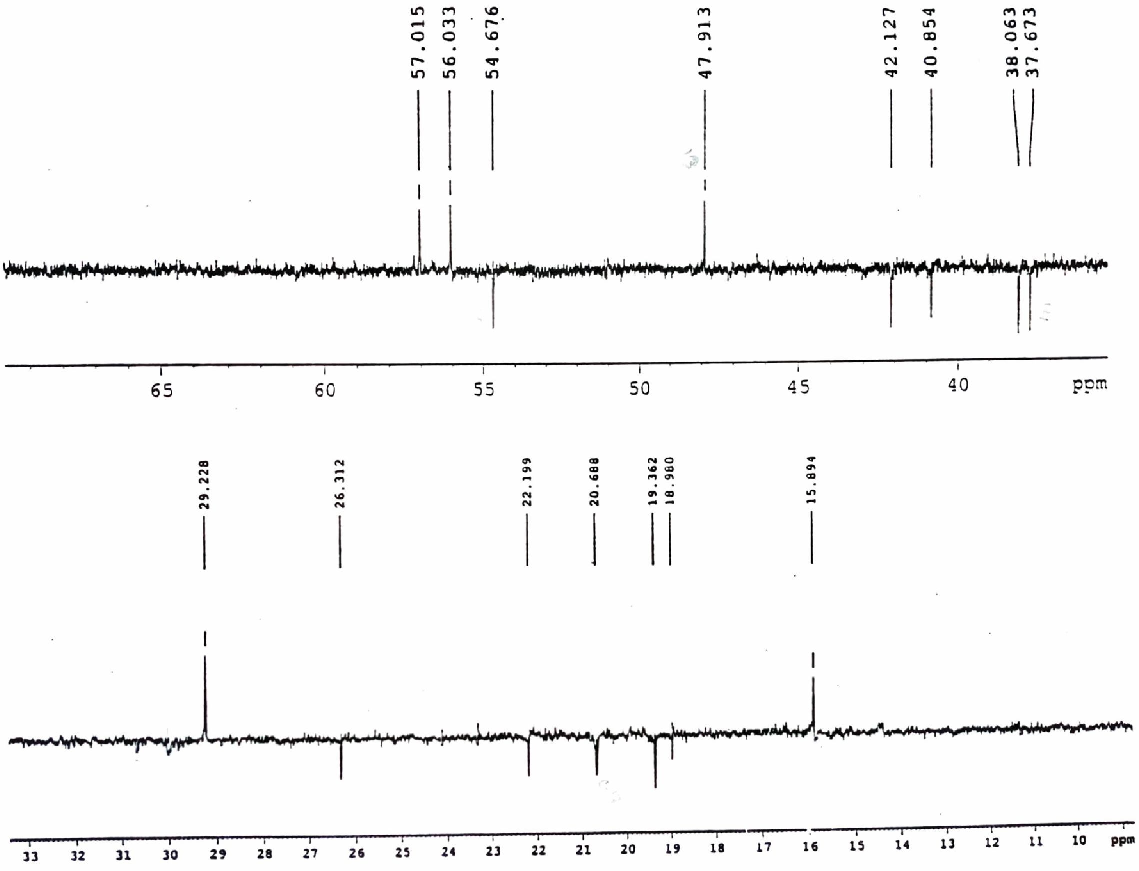

Figure S3.^{13}C NMR DEPT 135°(400 MHz, $CDCl_3$) spectrum of compound **1**.

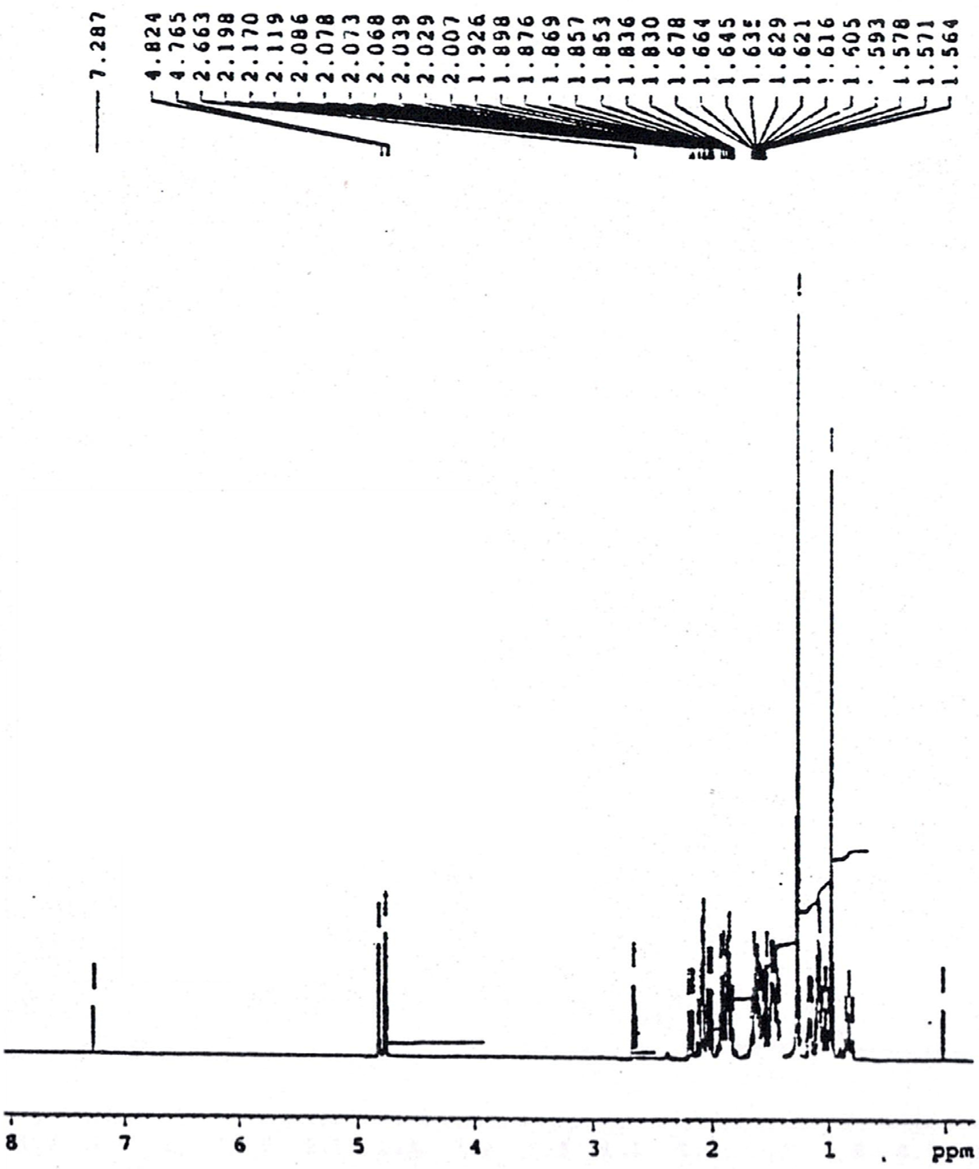

Figure S4. ^{1}H NMR (400 MHz, $CDCl_3$) spectrum of compound **2**.

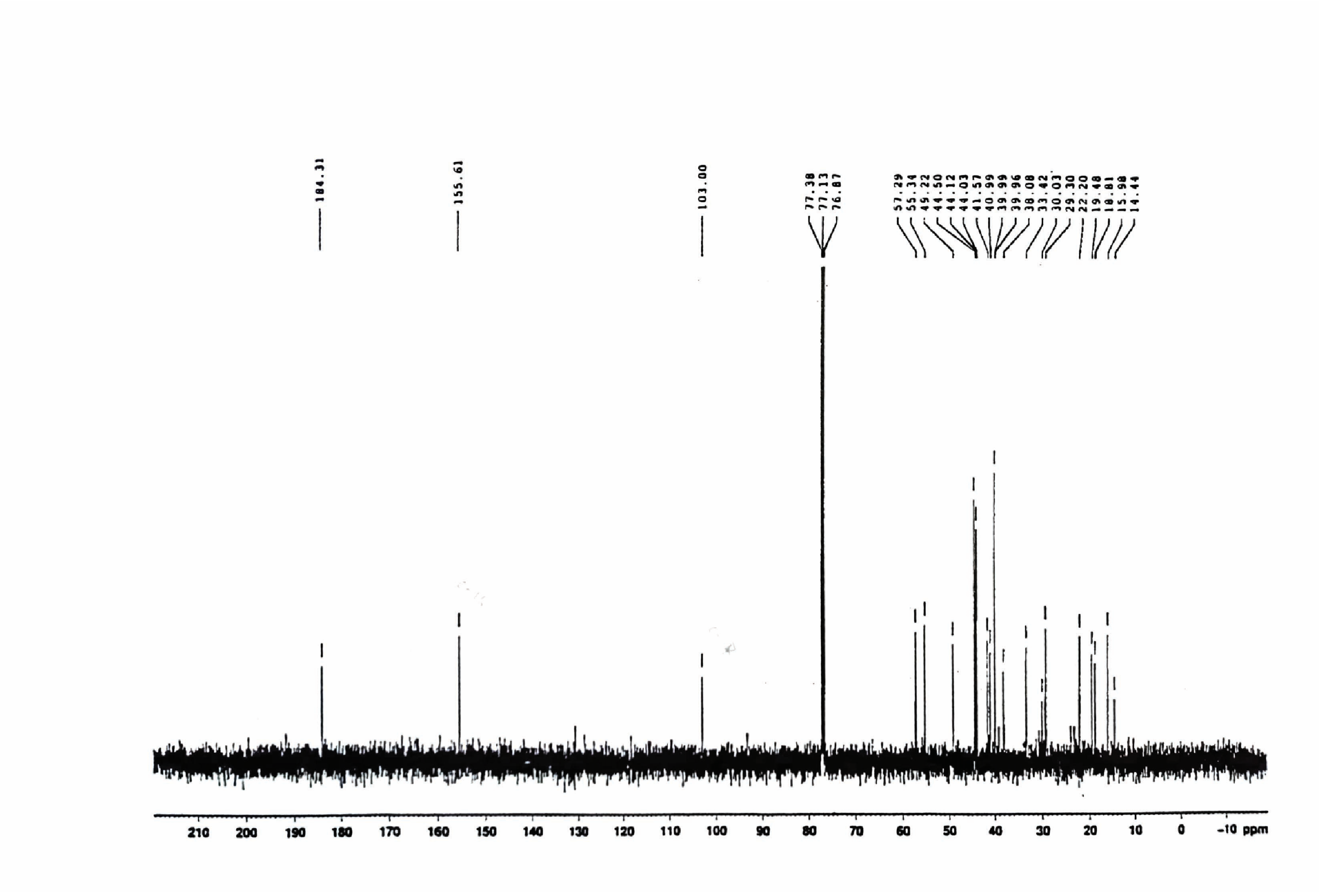

Figure S5.^{13}C NMR (400 MHz, $CDCl_3$) spectrum of compound **2**.

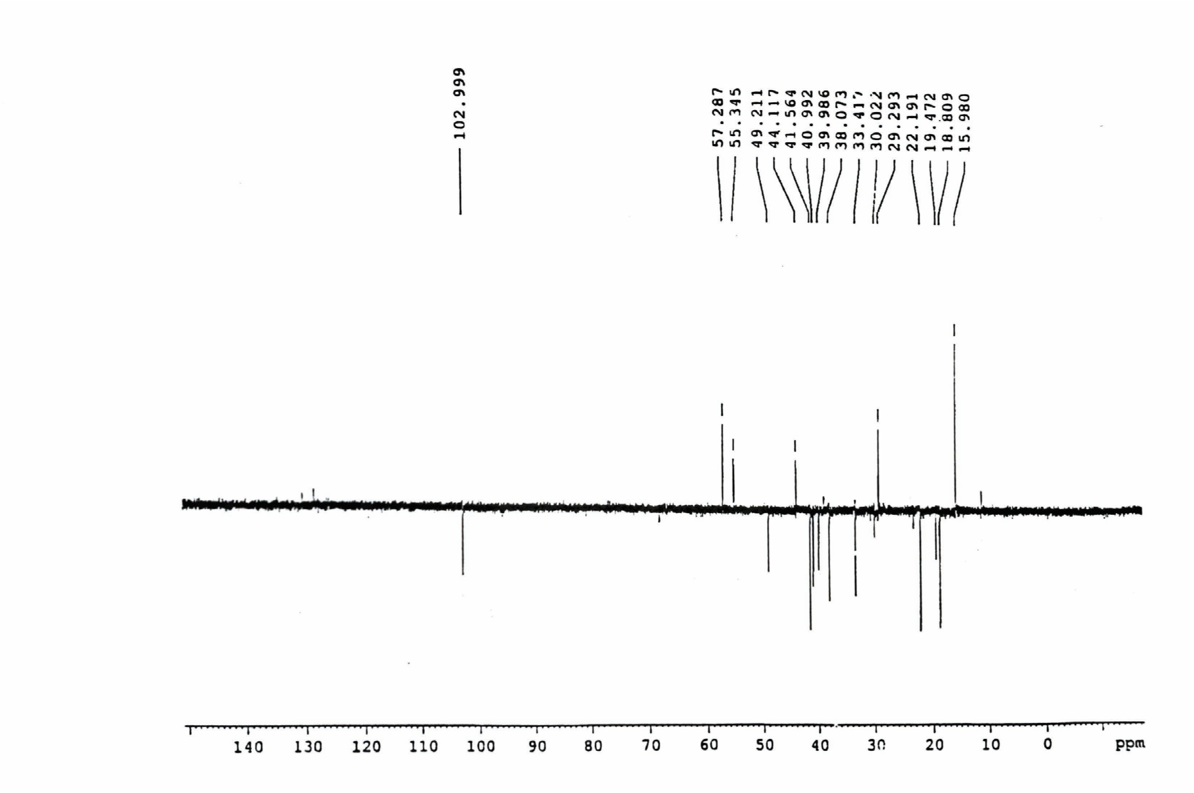

Figure S6.^{13}C NMR DEPT 135° (400 MHz, $CDCl_3$) spectrum of compound **2.**

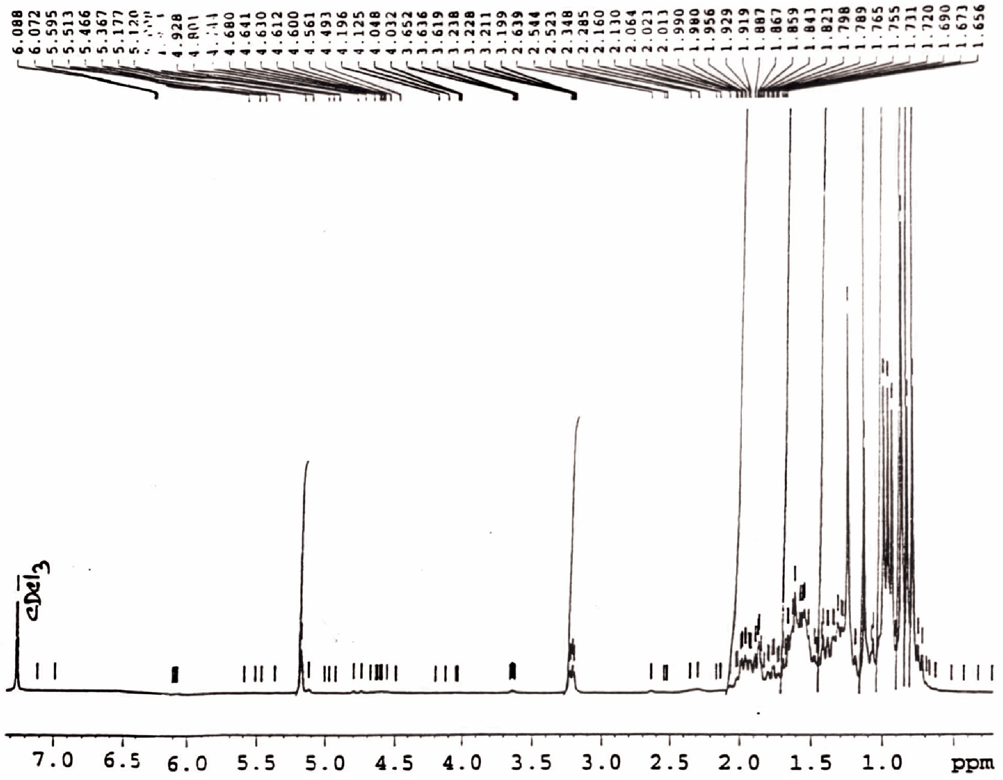

Figure S7.^{1}H NMR (400 MHz, $CDCl_3$) spectrum of compound **3**.

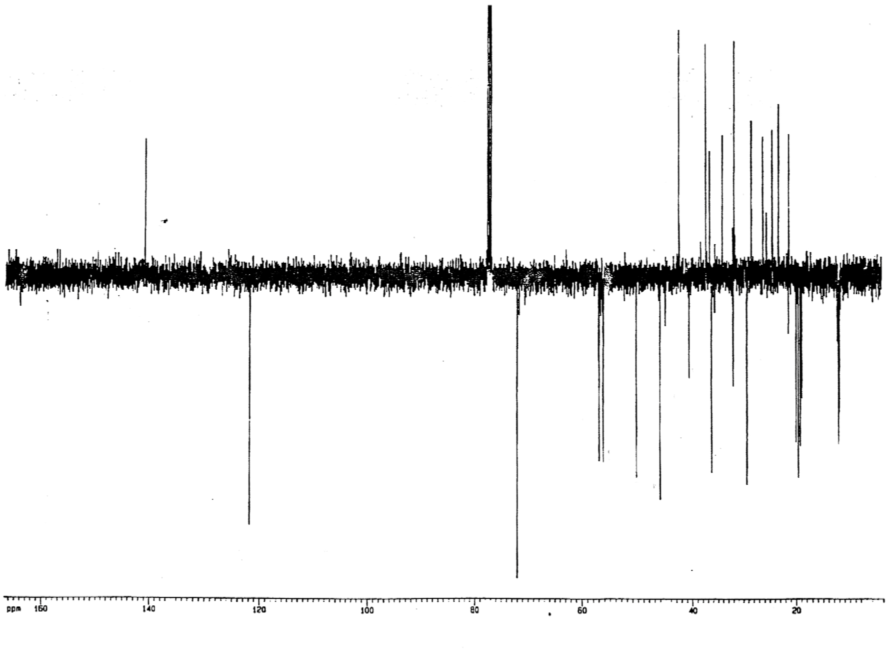

Figure S8.^{13}C NMR DEPT 135° (400 MHz, $CDCl_3$) spectrum of compound **3.**

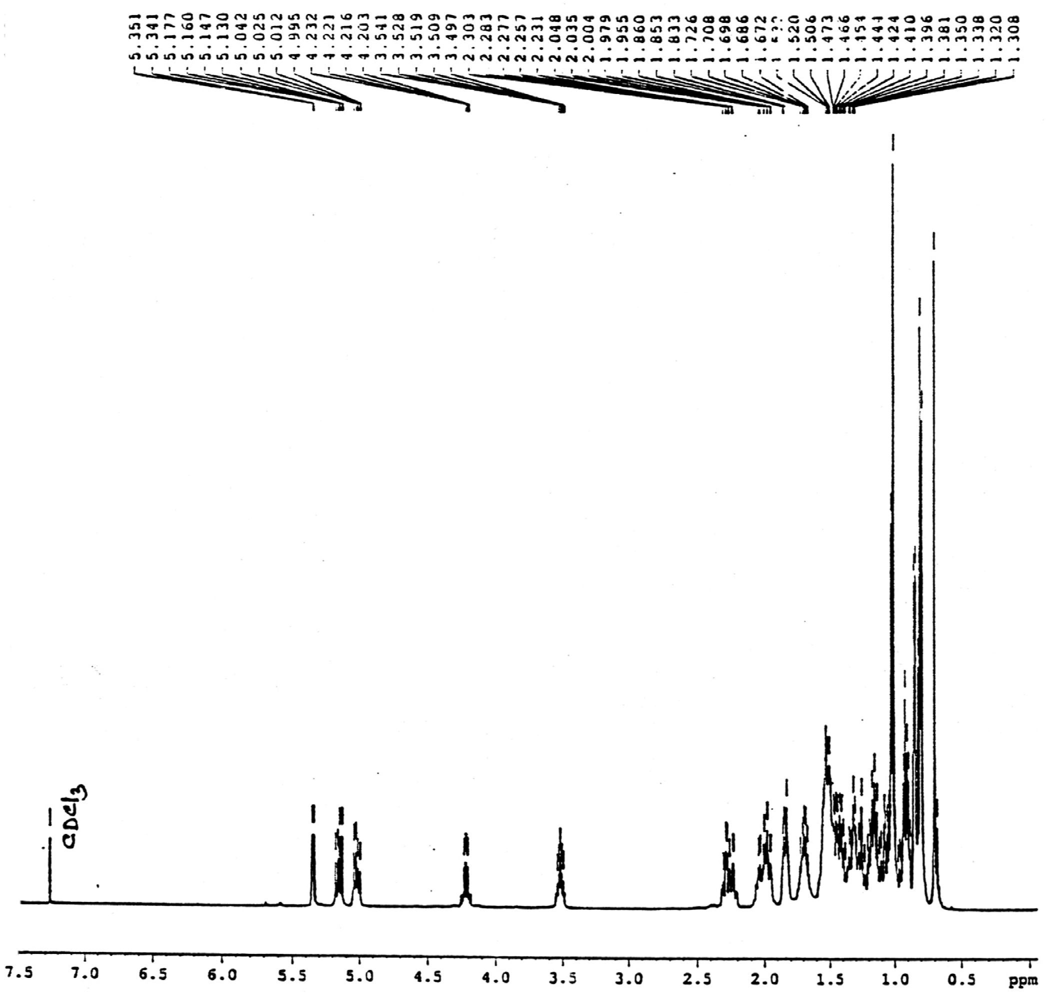

Figure S9. ^{1}H NMR (400 MHz, $CDCl_3$) spectrum of compound **4**.

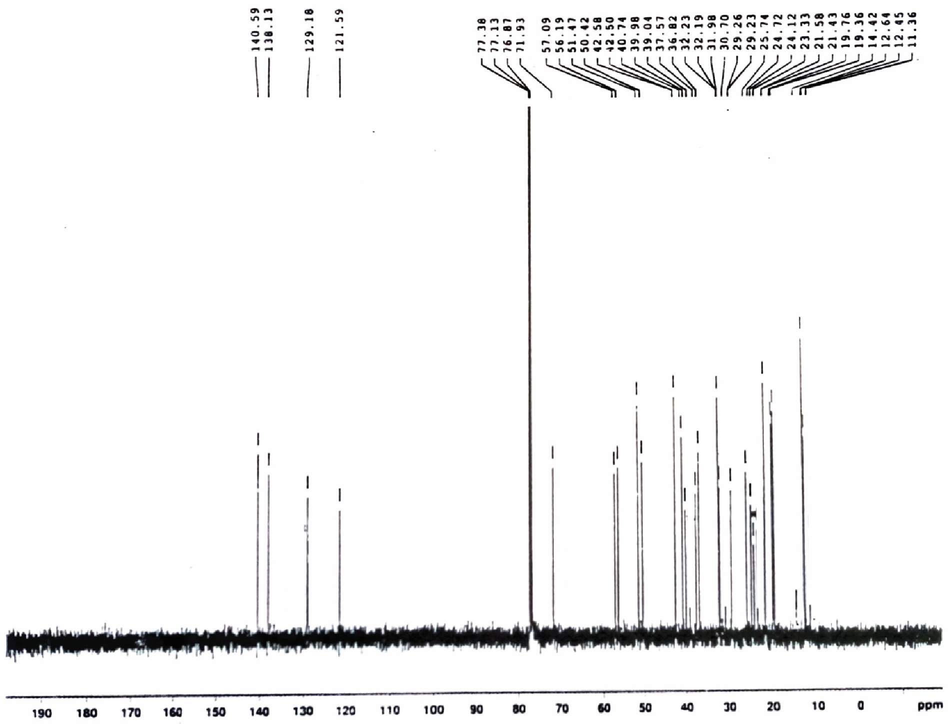

Figure S10. ^{1}H NMR (400 MHz, $CDCl_3$) spectrum of compound **4**.

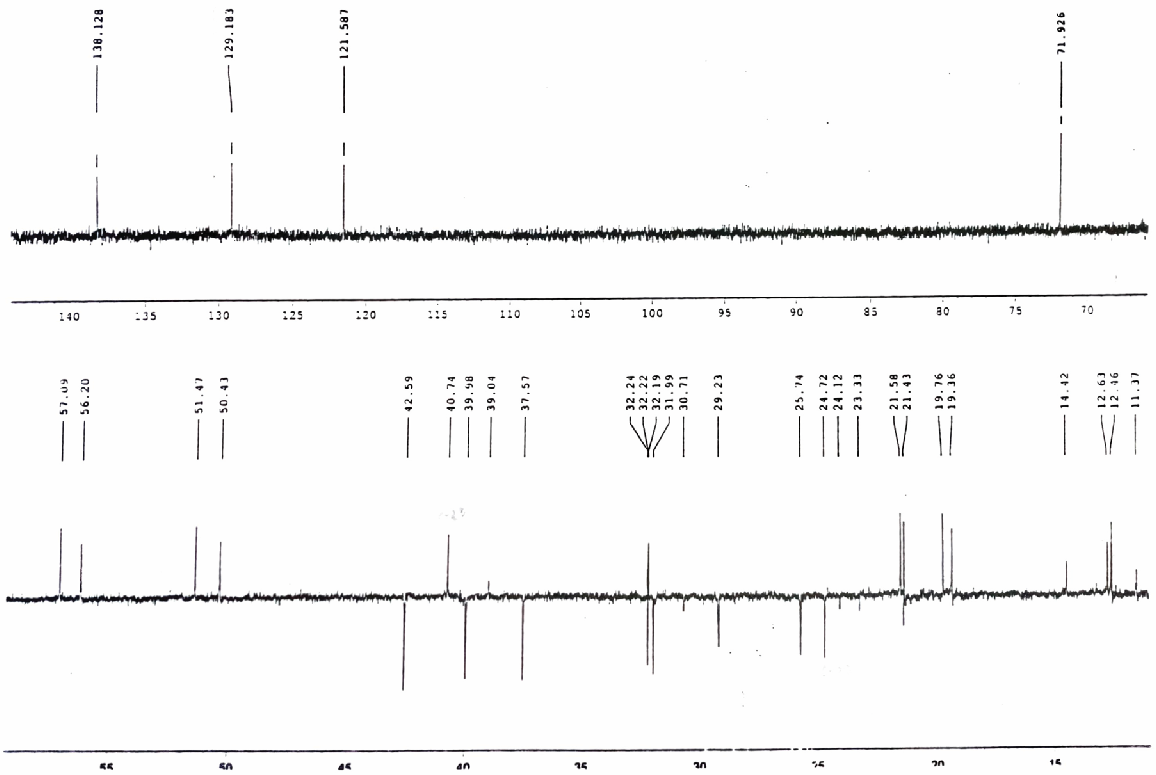

Figure S11. ^{13}C NMR DEPT 135° (400 MHz, $CDCl_3$) spectrum of compound **4.**
